# Hemodynamics in Cerebral Aneurysms and Parent Arteries With Incompletely Expanded Flow Diverter Stents

**DOI:** 10.1002/cnm.70033

**Published:** 2025-03-31

**Authors:** Soichiro Fujimura, Kazuya Yuzawa, Katharina Otani, Kostadin Karagiozov, Hiroyuki Takao, Toshihiro Ishibashi, Koji Fukudome, Makoto Yamamoto, Yuichi Murayama

**Affiliations:** ^1^ Department of Mechanical Engineering Tokyo University of Science Tokyo Japan; ^2^ Division of Innovation for Medical Information Technology The Jikei University School of Medicine Tokyo Japan; ^3^ Graduate School of Mechanical Engineering Tokyo University of Science Tokyo Japan; ^4^ Department of Neurosurgery The Jikei University School of Medicine Tokyo Japan; ^5^ Siemens Healthcare K.K Tokyo Japan; ^6^ Department of Mechanical Engineering Kanazawa Institute of Technology Nonoichi Ishikawa Japan

## Abstract

Braided stents for cerebral aneurysms, including flow diverter stent (FDS), may exhibit incomplete stent expansion (IncompSE) during deployment, depending on factors related to the parent artery. Poor stent apposition due to IncompSE can increase the risk of complications or incomplete aneurysm occlusion. Since hemodynamics may play a critical role in these adverse events, we investigated hemodynamic parameters associated with IncompSE using computational fluid dynamics (CFD) analysis. Three basic geometries were generated to represent an aneurysm located on the siphon of the internal carotid artery. CFD analysis was conducted for each geometry under a total of 12 patterns, including before deployment, complete stent expansion (CompSE), and IncompSE on the distal and proximal sides. We focused on hemodynamic parameters reported to influence occlusion or complications after FDS deployment. The change rate (CR) of these parameters was calculated by comparing conditions before and after FDS deployment. In the cases of CompSE, volume flow (VF) into the aneurysm and maximum wall shear stress (WSS) on the aneurysmal wall decreased on average by 52.7% and 34.7%, respectively. Conversely, in the cases of IncompSE, higher VF, inflow jets, and vortices were observed within the aneurysm. Increased WSS at the aneurysmal neck and parent artery was also noted. While static pressure on the aneurysmal wall and energy loss through the aneurysm region showed minimal change in the case of CompSE, both parameters increased in cases of IncompSE. These findings suggest that IncompSE may result in hemodynamic conditions that are suboptimal for treatment. IncompSE of FDS can potentially induce unfavorable hemodynamic changes, including increased blood flow into the aneurysm and elevated pressure on the aneurysmal wall compared to pre‐deployment conditions.

## Graphical abstract

Incomplete stent expansion (IncompSE) during flow diverter stent (FDS) deployment may lead to unfavorable hemodynamic changes, increasing risks of complications and incomplete aneurysm occlusion. Using computational fluid dynamics (CFD), we analyzed three basic aneurysm models under various incompSEs. IncompSE resulted in higher volume flow, wall shear stress, pressure, and energy loss compared to complete expansion. These findings suggest that IncompSE may create suboptimal hemodynamic conditions, potentially compromising the efficacy of FDS treatment for cerebral aneurysms.

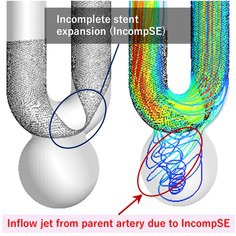

## 1 Introduction

An intracranial stent is one of the most commonly used devices in endovascular treatment of cerebral aneurysms. In particular, the flow diverter stent (FDS) reduces the risk of aneurysm rupture by altering the flow due to its high wire mesh density, induces neointima formation, and promotes thrombosis to occlude the aneurysmal sac [1, 2, 3, 4, 5, 6, 7]. Including the Pipeline Embolization Device (Covidien/Medtronic, Irvine, CA, USA), the first commercialized FDS approved by the FDA (Food and Drug Administration) in 2008, most FDSs are braided stents, consisting of wires braided together. Since braided stents are self‐expanding, with a degree of restorative force, incomplete stent expansion (IncompSE) may occur depending on the artery characteristics in which the FDS is deployed [8, 9, 10, 11, 12, 13, 14, 15, 16, 17]. When the expected flow diversion effect is not achieved due to the occurrence of IncompSE, the patient is at risk of aneurysm non‐occlusion, leading to unsuccessful treatment [18, 19]. In addition, it may lead to serious complications such as delayed rupture after the deployment and thromboembolic or ischemic events [11, 20, 21, 22, 23, 24, 25].

It has been proposed that hemodynamics may play an important role in aneurysm thrombosis formation and the occurrence of other postoperative complications [26, 27, 28, 29, 30, 31, 32]. Specifically, because the concept of the FDS is to treat aneurysms by altering blood flow, a number of investigations have been conducted using computational fluid dynamics (CFD) analysis under conditions of complete stent expansion (CompSE). Such studies include investigations of hemodynamic factors involved in outcomes after deployment and the “ideal” design of FDS [33, 34, 35]. Multiple reports have also been published on postoperative complications and hemodynamics after FDS deployment [21, 36, 37, 38]. However, studies investigating the hemodynamics in the case of IncompSE have been very limited [9, 39]. The identification of hemodynamic characteristics in the cases of IncompSE may help the surgeon to determine an optimal clinical strategy for patient management.

In this study, we conducted CFD analysis for aneurysms without stent, with CompSE, and with IncompSE. Multiple aneurysm types and multiple patterns of IncompSE were analyzed. Considering hemodynamic parameters reported to be involved in aneurysm occlusion and postoperative complications after FDS deployment, we aimed to investigate how the occurrence of IncompSE affects the hemodynamics.

## 2 Materials and Methods

### 2.1 Basic Model of Artery and Aneurysm

**FIGURE 1:**
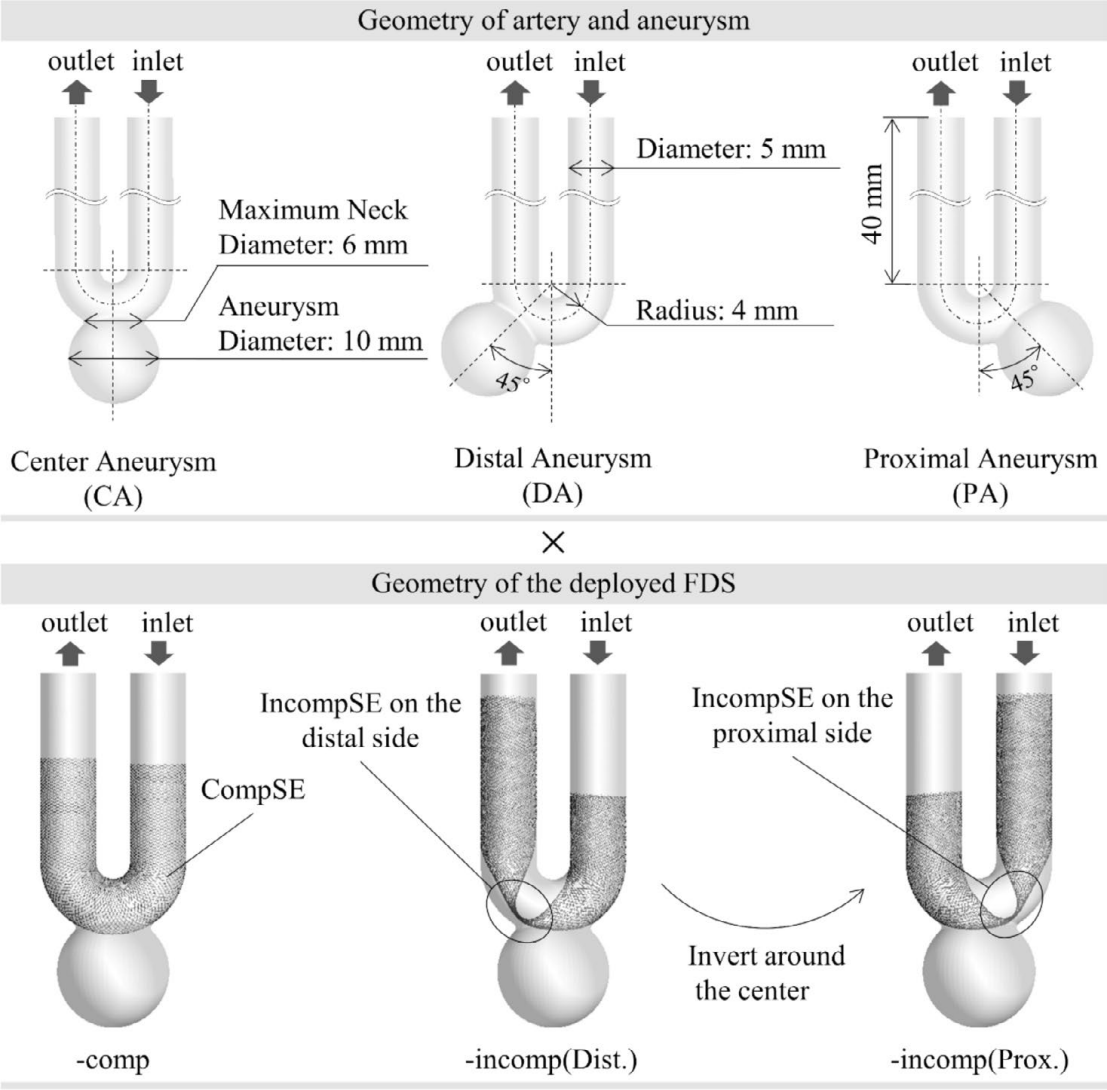
Generated three types of basic aneurysms and deployed FDS. The central aneurysm (CA), distal aneurysm (DA), and proximal aneurysm (PA) models were combined with complete stent expansion (‐comp), incomplete stent expansion on the distal side (‐incomp(Dist.)), and incomplete stent expansion on the proximal side (‐incomp(Prox.)).

Three types of basic artery and aneurysm models were generated to imitate a large aneurysm located on the “siphon” of the internal carotid artery (ICA). The parent artery, with a diameter of 5 mm, executed a 180° turn with a radius of 4 mm for the centerline curvature to imitate the siphon. At the siphon, the aneurysm was simulated at the center of the curvature in the center aneurysm (CA) model. The other two aneurysms were simulated at 45° to the center of the siphon on the distal and proximal sides in the distal aneurysm (DA) and the proximal aneurysm (PA) models, respectively. The lengths of the inlet and outlet segments were both 40 mm. The geometries at the aneurysm were the same, with a dome diameter of 10 mm and a maximum neck diameter of 6 mm. These three‐dimensional geometries of the basic model were generated using the CAD software, ZW3D package (ZWCAD Software Co. Ltd., Guangzhou, China). The details of the models were illustrated in Figure 1.

### 2.2 Modelling of Deployed FDS

We virtually deployed a FDS simulating the pipeline embolization device (PED) (Covidien/Medtronic, Irvine, CA, USA) sized 5 × 30 in the parent artery of the basic model. Regarding the geometry of the deployed FDS, we generated two types of apposition: CompSE and IncompSE. The stent geometry of CompSE was generated using our validated original virtual stent deployment simulation software technique [34, 40, 41, 42]. More specifically, the centerlines were segmented into 0.1 mm increments to determine the average diameter of the artery at these specific points by creating a section perpendicular to the centerline at each point. By referencing the centerlines and the corresponding diameters at each location, a virtual stent was deployed considering its maximum expansion diameter and length in that environment. This method allows for ideal stent deployment with no gap between the stent and the parent artery, representing adequate stent apposition. This situation was labeled as “‐comp” (i.e., complete). In contrast, the geometry of IncompSE was obtained through stent deployment simulation using FEM‐based structural analysis. The structural analysis was conducted using the finite element analysis software Abaqus/Explicit ver. 6.14 (Dassault Systèmes Simulia Corp., Providence, RI, USA). The stent deployment simulation was conducted based on a validated method developed by Ma et al. [43]. The following three steps were conducted: (i) Stent crimping: The stent is radially compressed and housed within a microcatheter. (ii) Stent bending: The microcatheter containing the stent is bent according to the parent artery shape. (iii) Stent deployment: A rigid wall in the lumen, which prevents passing, is placed at the proximal stent end in the microcatheter. The microcatheter is then withdrawn along the centerline of the vascular model, allowing the stent to be deployed through the rigid wall. The stent wires were modeled as three‐dimensional Timoshenko beam elements. A mesh size of 0.09 mm was used, and the material properties for the stent were assigned based on cobalt–chromium alloy characteristics (Young's modulus of 206 GPa, yield stress of 2.8 GPa, isotropic hardening slope of 8.8 GPa, and density of 8 g/cm^3^). The microcatheter was modeled as a hollow cylinder with shell elements, having an inner diameter of 0.53 mm and a thickness of 0.080 mm. The material properties of the microcatheter were set with a Young's modulus of 1 GPa, a Poisson's ratio of 0.49, and a density of 1 g/cm^3^. General contact based on the penalty method in Abaqus/Explicit was employed for contact interactions. The friction coefficients between the wires, microcatheter, and vascular model were set at 0.2, 0.05, and 0.3, respectively, while the friction coefficient between the microcatheter and the vascular model was set at 0.1. In the FDS deployment procedure in step (iii), the IncompSE was intentionally reproduced in the deployment at the parent artery to obtain its geometry. Since the IncompSE could occur in two ways, on both sides from the center of the U‐shape tip in the basic model, we considered two patterns of the IncompSE, one occurring on the proximal side, labeled as “‐incomp(Prox.),” and the other on the distal side, labeled “‐incomp(Dist.),” applying both of them to the three basic models. The three‐dimensional geometries of the deployed FDSs were generated in a configuration in which each constituent wire was individually represented.

### 2.3 Hemodynamic Analysis Using CFD

**FIGURE 2:**
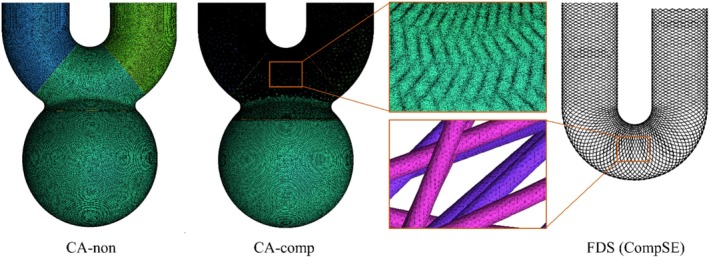
Examples of the generated computational mesh. The CA‐non and CA‐comp were selected as illustrative cases, with meshes around the FDS wires also displayed.

Considering the three types of basic aneurysm models and the deployed stent, we conducted CFD analysis for a total of 12 patterns, including CA‐non, CA‐comp, CA‐incomp(Dist.), CA‐incomp(Prox.), DA‐non, DA‐comp, DA‐incomp(Dist.), DA‐incomp(Prox.), PA‐non, PA‐comp, PA‐incomp(Dist.), PA‐incomp(Prox.). The computational mesh was generated using ANSYS ICEM CFD (ANSYS Inc., Canonsburg, PA, USA). In the case of “non‐stent” aneurysm (i.e., before the stent deployment), a seven‐layer prism mesh with a height of 0.3 mm was arranged in the vicinity of the artery and a tetrahedral mesh was generated in the lumen. The maximum mesh size was set to 0.15 mm for the aneurysm and the parent artery, and 0.3 mm for all other regions. The total number of elements was approximately 5.5 million. In contrast, for aneurysms with deployed FDS, only a tetrahedral mesh was arranged at the stented section of the artery, while prism mesh was arranged near the aneurysm wall, as well as the proximal and distal regions of the parent artery relative to the stent. The mesh size around the wires of the FDS was set to 0.008 mm, while the mesh size on the wall of the parent artery where the FDS was deployed was set to 0.1 mm. After the FDS deployment, the total number of elements was approximately 225 million on average. The generated computational mesh for the CA‐non and CA‐comp was displayed in Figure 2 as illustrative cases. Grid independence studies were performed in previous studies to demonstrate that the grid size was sufficient to obtain results independent of the grid size [44, 45, 46]. To apply the fully developed flow, 75 mm extended tubes were connected to inlets and outlets. The flow field was assumed to be incompressible laminar flow since the Reynolds number based on the artery of 5 mm diameter was 245. Blood was modeled as a Newtonian fluid with a density of 1100 kg/m^3^ and a viscosity of 0.0036 Pas, respectively [34, 44]. The walls of the artery and stent were assumed to be rigid bodies, and a nonslip boundary condition was imposed. As a solver, ANSYS CFX 2020 R1 was used. A steady flow simulation was conducted by imposing 0.003465 kg/s as the inlet boundary condition, which corresponds to the measured diastolic flow in healthy adults as reported by Ford et al. [47]. The static pressure (SP) fixed at 0 Pa was imposed as the outlet boundary condition. These boundary conditions have been validated and used in previous studies [44, 48, 49].

### 2.4 Evaluation Parameters

To evaluate the flow in the arterial model, we focused on several hemodynamic parameters reported as contributing factors to occlusion or complication after FDS deployment [26]. Among these parameters, the maximum wall shear stress (WSS_max_) and the average SP were calculated as the forces acting on the wall. Since the main concept of the FDS is to reduce flow into the aneurysm, the volume flow (VF) into the aneurysm at its neck was also evaluated. The energy loss (EL) was an energy‐based parameter. This parameter was developed to evaluate the EL when blood passes through an artery with an aneurysm [29, 44, 50]. A higher value indicates greater resistance to blood flow, reflecting increased flow irregularity. The parameter was defined as the following equation:

(1) $$\mathrm{EL}=v_{\text{in}}A\left\{\left(\frac{1}{2}{\mathrm{\rho v}}_{\text{in}}^{2}+P_{\text{in}}\right)-\left(\frac{1}{2}{\mathrm{\rho v}}_{\text{out}}^{2}+P_{\text{out}}\right)\right\}$$

where *A* is the area of the cross‐sectional plane of the parent artery, *v* is the averaged velocity, *P* is the averaged static pressure, and subscripts “in” and “out” indicate the inlet and outlet plane of the model, respectively. Since the basic model was adopted in the present study, no differences in EL due to the parent artery were expected to occur. Therefore, while EL is usually calculated around an aneurysm, the present EL was obtained from the entire analysis domain. In addition, to identify the flow change between before and after stent deployment, the change rate (CR) was defined as:

(2) $$\begin{array}{c} \mathrm{CR}=\frac{X_{\text{post}}-X_{\mathrm{pre}}}{X_{\mathrm{pre}}}\; \end{array}$$

where *X*_pre_ and *X*_post_ indicate the parameters before and after stent deployment, respectively.

## 3 Results

### 3.1 Flows After Stent Deployment in CompSE and IncompSE

**FIGURE 3:**
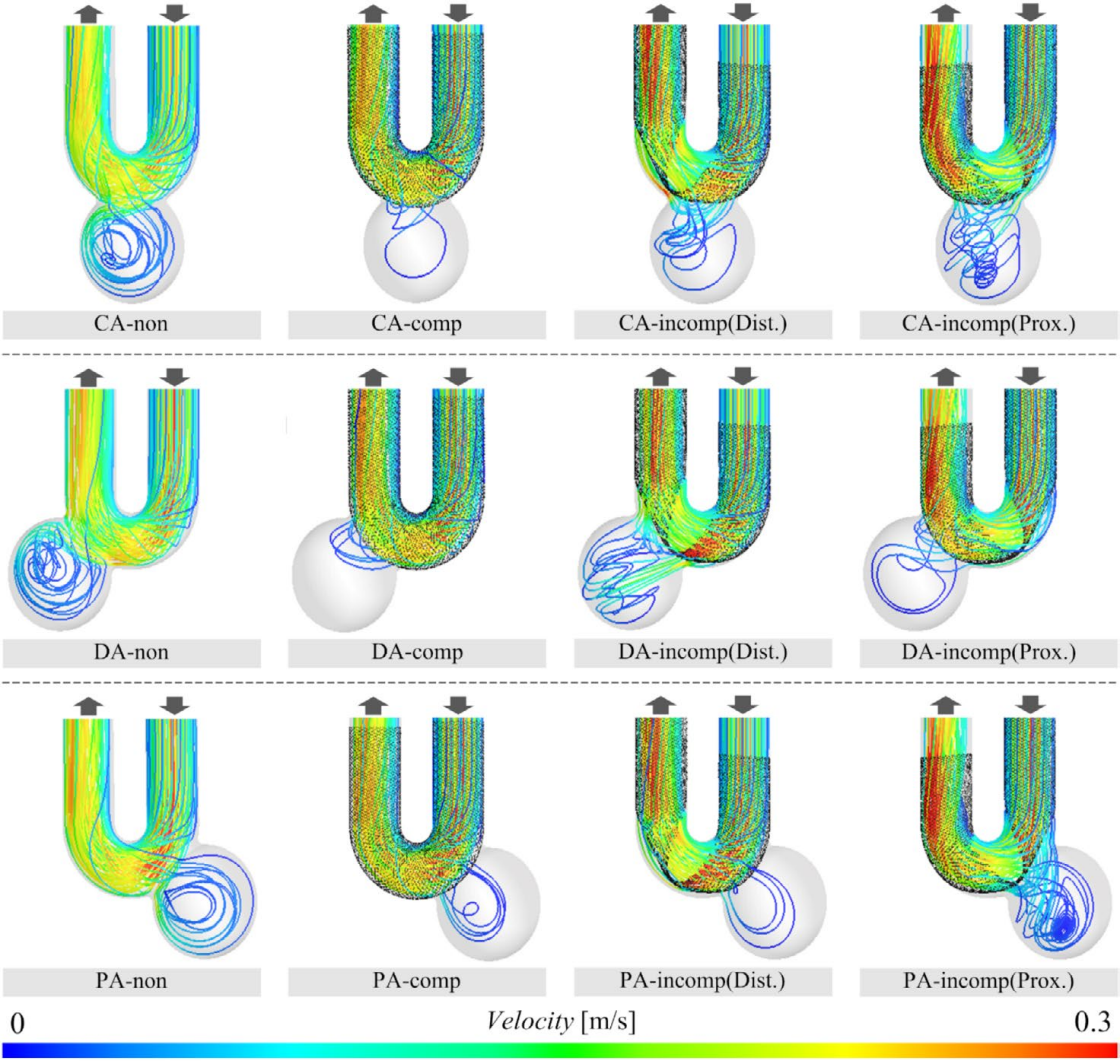
Streamlines in the cases of before FDS deployment, CompSE, and IncompSE. The streamlines are colored in the range of 0–0.3 m/s according to the velocity.

**FIGURE 4:**
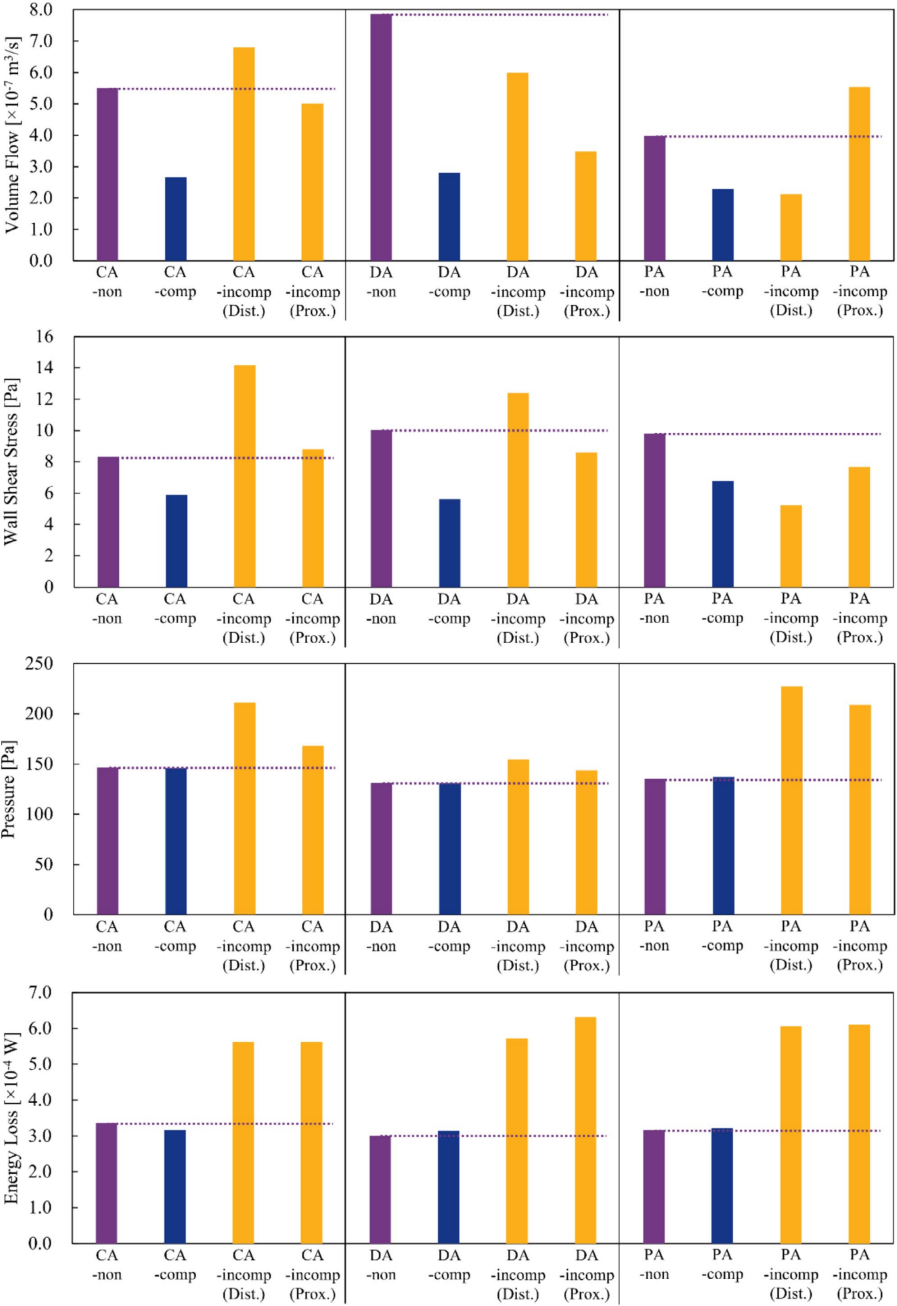
The values of hemodynamic parameters for each analysis case. The dashed line is to compare the values after FDS deployment with the value before that.

The streamlines in each case obtained as a result of CFD analysis are shown in Figure 3. In addition, the detailed values of the parameters and CR are summarized in Table 1 and Figure 4. It should be noted that the pressure does not represent a real physiological value but indicates the pressure relative to the outlet pressure. In the case before FDS deployment, although the amount of blood inflow into the aneurysm varied depending on the type of aneurysm, we identified flow along the aneurysmal wall, from the inflow at the neck to the outflow to the parent artery, forming a primary vortex regardless of the type of aneurysm. The values of VF for CA‐non, DA‐non, and PA‐non were 5.50 × 10^−7^, 7.86 × 10^−7^, and 3.98 × 10^−7^ m^3^/s, respectively. In addition, the ELs were 3.36 × 10^−4^ W for CA‐non, 3.01 × 10^−4^ W for DA‐non, and 3.16 × 10^−4^ W for PA‐non, respectively, with no significant differences among the aneurysm types.

**TABLE 1:** The value of the investigated hemodynamic parameters in each case and CR in the cases of CompSE and IncompSE.

Parameters	Cases	CA	DA	PA
VF [×10^−7^ m^3^/s]	‐non	5.50	7.86	3.98
	‐comp	2.66	2.81	2.30
	CR [%]	−51.6%	−64.2%	−42.2%
	‐incomp(Dist.)	6.80	5.99	2.13
	CR [%]	23.6%	−23.8%	−46.4%
	‐incomp(Prox.)	5.01	3.48	5.54
	CR [%]	−8.90%	−55.8%	39.4%
WSSmax [Pa]	‐non	8.32	10.1	9.79
	‐comp	5.89	5.63	6.77
	CR [%]	−29.2%	−44.0%	−30.9%
	‐incomp(Dist.)	14.17	12.37	5.22
	CR [%]	70.3%	23.1%	−46.7%
	‐incomp(Prox.)	8.79	8.59	7.66
	CR [%]	5.56%	−14.6%	−21.8%
SP [Pa]	‐non	147	131	135
	‐comp	146	131	137
	CR [%]	−0.29%	−0.2%	1.58%
	‐incomp(Dist.)	212	154	227
	CR [%]	44.3%	17.7%	68.1%
	‐incomp(Prox.)	168	144	209
	CR [%]	14.6%	9.66%	54.5%
EL [×10^−4^ W]	‐non	3.36	3.01	3.16
	‐comp	3.17	3.14	3.22
	CR [%]	−5.80%	4.47%	1.67%
	‐incomp(Dist.)	5.62	5.72	6.06
	CR [%]	67.4%	90.2%	91.5%
	‐incomp(Prox.)	5.62	6.31	6.11
	CR [%]	67.4%	110%	92.9%

In contrast, when the FDS was completely expanded, we qualitatively and quantitatively identified a reduction in inflow into the aneurysm compared to before deployment; the CR of VF ranged from −64.2% for DA to −42.2% for PA, indicating a decrease for all types of aneurysms. As in the cases before FDS deployment, the ELs did not differ significantly among the aneurysm types (the values were 3.17 × 10^−4^, 3.14 × 10^−4^, and 3.22 × 10^−4^ W for CA‐comp, DA‐comp, and PA‐comp, respectively). Furthermore, the CR in EL before and after deployment remained below 6% in all cases.

In contrast, in the cases of IncompSE, we observed complex flow in the aneurysm, as seen in CA‐incomp(Prox.) and PA‐incomp(Prox.). In DA‐incomp(Dist.), an inflow jet from the parent artery to the aneurysmal sac was observed. Among cases of IncompSE, the CRs in VF showed both increases and decreases, ranging from −55.8% to +39.4%. The ELs increased in all types of aneurysms compared to before deployment, with CRs between +67.4% and +110%.

### 3.2 Change of the Force Acting on the Wall of Aneurysm and Artery

**FIGURE 5:**
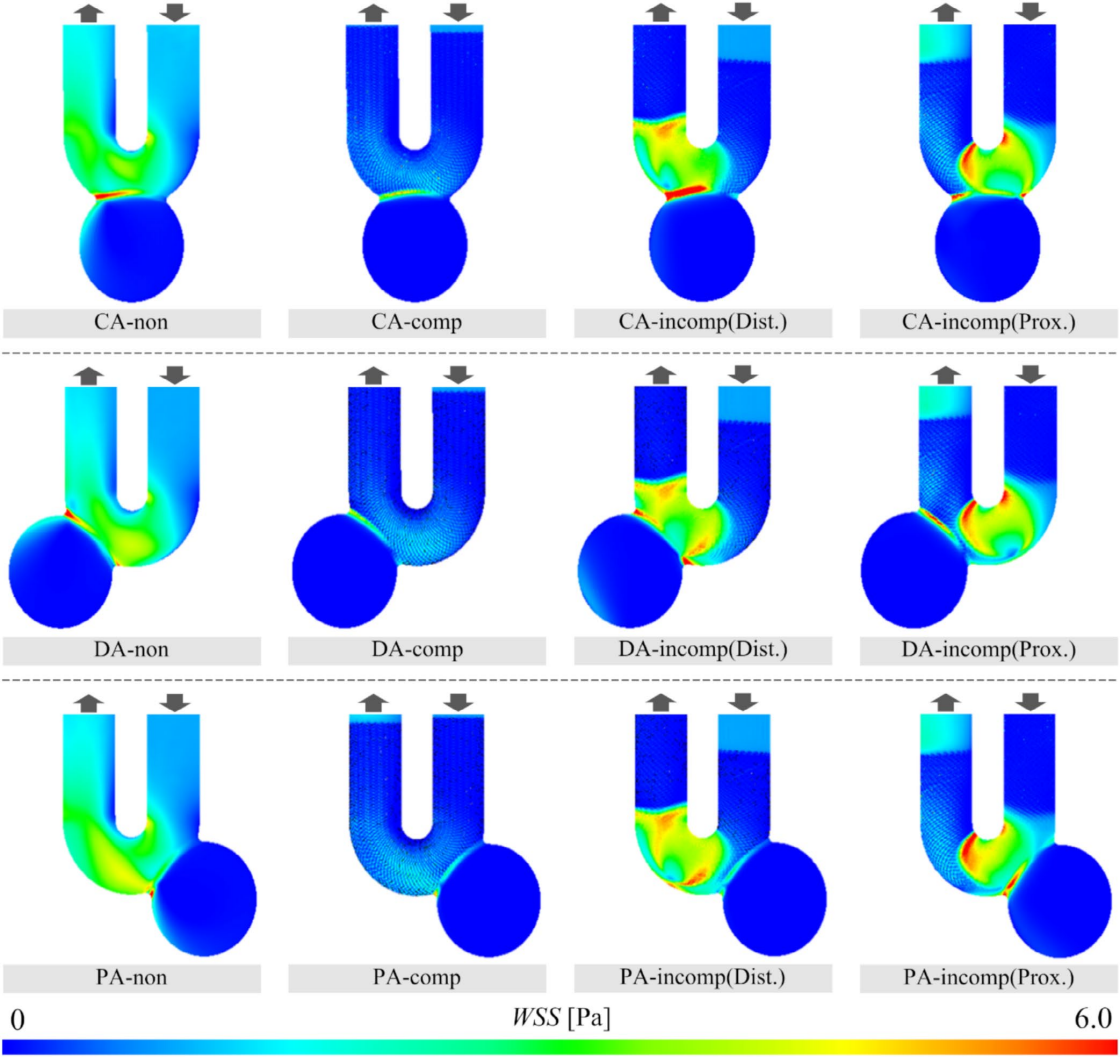
WSS on the wall in the cases before FDS deployment, CompSE, and IncompSE. The wall is colored in the range of 0–6.0 Pa.

**FIGURE 6:**
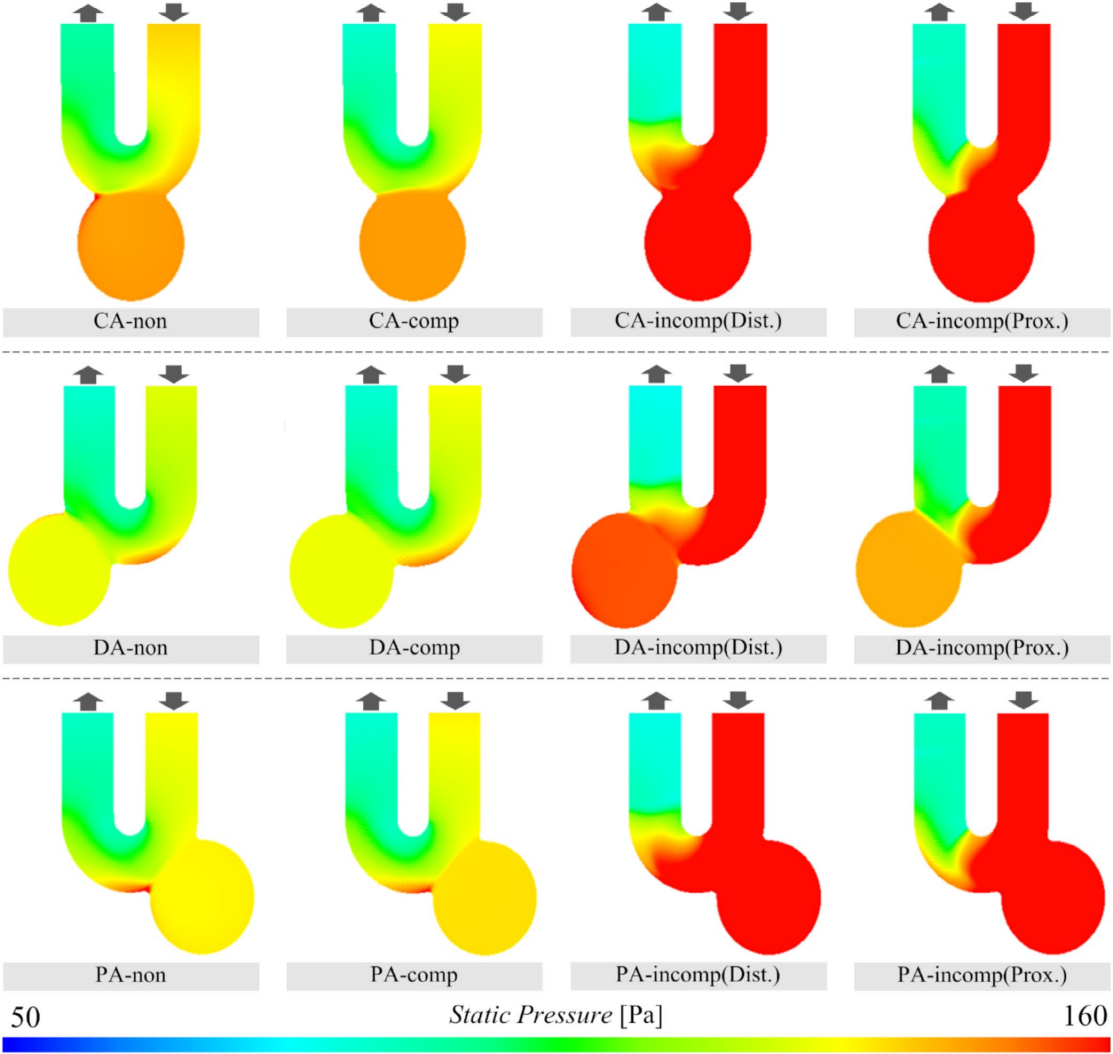
Static pressure on the wall in the cases before FDS deployment, CompSE, and IncompSE. The static pressure is colored in the range of 50–160 Pa.

The results of WSS and SP obtained from the CFD analysis are illustrated in Figures 5 and 6, respectively. Regarding WSS, it was observed to be higher on the distal side of the aneurysm neck for all three types of aneurysm models before the FDS deployment. The WSS_max_ on the aneurysm wall varied from 8.32 to 10.1 before deployment. The WSS_max_ in the case of CompSE ranged from 5.63 to 6.77, indicating a decrease after FDS deployment (the CR was between −29.2% and −44.0%). In addition, we qualitatively confirmed that WSS decreased in the parent artery after FDS deployment.

In contrast, when IncompSE occurred, WSS decreased in some cases and increased in others. Specifically, when IncompSE occurred on the distal side, the CRs for the CA and DA models were 70.3% and 23.1%, respectively. In contrast, in the PA‐incomp(Dist.) model, the WSS was decreased and CR was −46.7%. As for the cases of IncompSE on the proximal side, the CR decreased to −14.6% and − 21.8% in the DA and PA model, respectively. Although WSS increased in the CA model, the CR was 5.56%, indicating a relatively small change. In addition, in all cases of IncompSE, an increase in WSS in the parent artery was observed compared to before deployment.

Focusing on SP, the value of the CA model was higher than that of the other two models before FDS deployment. Even after the FDS was fully deployed (CompSE), there was little change in SP before and after deployment. In contrast, SPs were observed to increase compared to before deployment in both IncompSEs, indicating an increase in pressure on the wall of the aneurysm. The CR ranged from 9.66% to 68.1%. It was also qualitatively confirmed that SP increased in the parent artery upstream of the location where IncompSE occurred, compared to before deployment.

## 4 Discussion

### 4.1 Effect of Hemodynamic Changes due to IncompSE

Among braided stents, the FDS has a high mesh density to achieve a flow diversion effect; therefore, the effect of IncompSE on blood flow must be considered significant. In fact, the results of this study revealed significant differences in hemodynamics between CompSE and IncompSE. Furthermore, quantitative evaluation of hemodynamic parameters indicated alterations that might be adverse to the treatment of aneurysms (e.g., hemodynamic changes that may cause incomplete occlusion of the aneurysm or complications such as delayed rupture). Hemodynamic investigations of FDS using CFD analysis have focused mainly on blood inflow into cerebral aneurysms, forces acting on the wall surface, and flow energy. Therefore, in this study, we followed the same focus and set the evaluation parameters of VF, WSS, SP, and EL, which have been frequently investigated in previous studies [26].

### 4.2 VF Into Aneurysm

The main concept of the FDS is to occlude the aneurysm by altering the flow, promoting aneurysm thrombosis and the formation of neointima [3, 4, 5]. The presented VF is defined to evaluate blood flow into the cerebral aneurysm. In fact, in the cases of CompSE, the CR of VF averaged −52.7%, confirming that the stent mesh effectively diverted blood flow in accordance with its concept. In contrast, in the cases of IncompSE, the CR of VF increased in 2 out of 6 patterns. Specifically, it increased by 23.6% in CA‐incomp(Dist.) and by 39.4% in PA‐incomp(Prox.). In particular, referring to the streamlines in PA‐incomp(Prox.), the blood inflow into the aneurysm is greater than before FDS deployment, and the flow within the aneurysmal sac becomes more complex. Previous studies using patient‐specific aneurysm geometries have shown that IncompSE on the proximal side, counterintuitively, reduced the inflow into the aneurysm [9]. However, the present results indicate that depending on the location of the IncompSE and the aneurysm neck orifice, blood flow into the cerebral aneurysm may increase. Although the CR decreased in CA‐incomp(Prox.), the change was −8.90%, indicating that the flow diversion effect of stenting was not achieved as expected. In DA‐incomp(Dist.), the CR was −23.8%; however, the inflow jet into the aneurysm was confirmed with streamlines. Concentrated flow into aneurysms has been reported as a factor contributing to the occurrence of incomplete occlusion [29, 51]. In addition, some reports have implicated inflow jets as a cause of hemorrhagic complications after FDS deployment [52]. Therefore, such flow alterations induced by IncompSE may play a significant role in treatment outcomes after FDS deployment. In contrast, in some cases, such as PA‐incomp(Dist.), the flow diversion effect is comparable to that in CompSE (with CRs of −46.4% and − 42.2% for PA‐incomp(Dist.) and PA‐comp, respectively). In this case, the aneurysm was located on the proximal side, while the IncompSE occurred on the distal side. Thus, the FDS covered the neck of the cerebral aneurysm, achieving the expected flow diversion effect. Our present findings indicate that depending on the location of the IncompSE and the position of the aneurysm neck orifice, not only may the flow diversion effect be insufficient, but blood flow into the cerebral aneurysm may even increase.

### 4.3 Wall Shear Stress on Aneurysm Wall

Similar to the results for VF, WSS on the aneurysm wall was also reduced in CompSE regardless of the aneurysm type. Additionally, the decreased WSS in the parent artery is considered to result from reduced blood flow friction due to good stent apposition. In contrast, when IncompSE occurred, the CR of WSS increased in three out of six patterns. Specifically, in CA‐incomp(Dist.), the CR was 70.3%, representing the largest increase among all patterns. The WSS visualization figure also shows that, in this case, the area of high WSS at the neck was larger compared to before FDS deployment. The narrowing of the FDS lumen caused by IncompSE is thought to have accelerated blood flow, resulting in increased WSS by impinging on the neck on the distal side (see the streamlines of CA‐incomp(Dist.) in Figure 3). In contrast, in the PA‐incomp(Dist.) case, WSS decreased more than in the CompSE case (PA‐comp). This may be because, as mentioned in the VF results above, the location of the IncompSE was on the distal side from the neck orifice. In summary, in all IncompSE cases, WSS in the parent artery was elevated compared to before deployment. In particular, WSS was higher at the locations where gaps between the parent artery and the stent occurred due to IncompSE. Although the relationship between WSS and the pathology of cerebral aneurysms has been widely discussed, some studies suggest that high WSS is associated with inflammation of the vessel wall [53, 54, 55]. In addition, several studies have linked thrombus formation and plaque progression to WSS [56, 57]. Therefore, the surgeon should keep in mind that the occurrence of IncompSE may lead to an increase in WSS, potentially affecting the postoperative outcome.

### 4.4 SP on Aneurysm Wall

The SP, a parameter that evaluates the pressure on the aneurysm wall, changed little before and after FDS deployment in cases of CompSE (i.e., the percentage change remained within ±2% across all cases). In contrast, in the cases of IncompSE, the pressure increased in all cases, averaging 34.8%. Increased pressure on the wall of aneurysms has been reported to be associated with the development of thin‐walled regions and eventual aneurysmal rupture. In particular, Suzuki et al. reported that the location of the maximum pressure during pulsation corresponds to the thin‐walled region, based on an analysis of 50 unruptured middle cerebral artery (MCA) aneurysms [58]. Furthermore, they suggested that the high‐pressure points, in addition to low WSS, might be useful for identifying rupture points [59]. In addition, pressure has also been reported to contribute to delayed rupture after FDS deployment [21, 36]. From these results, the pressure increase induced by IncompSE may potentially lead to delayed rupture. Among the IncompSE cases analyzed in this study, CA‐incomp(Dist.), PA‐incomp(Prox.), and PA‐incomp(Dist.) showed larger pressure increases than the other three cases (with CRs of 44.3%, 68.1%, and 54.5%, respectively). In particular, for PA‐incomp(Prox.), as mentioned in the discussion on VF, the blood inflow into the cerebral aneurysm increased due to the occurrence of IncompSE, and the flow within the aneurysm became more complex. In other words, in IncompSE cases such as PA‐incomp(Prox.), the incomplete expansion during FDS deployment may negatively impact the treatment of aneurysms and lead to potential complications.

### 4.5 EL Through the Stent Deployed Artery and Aneurysm

Previous studies have often compared EL between successful and unsuccessful cases of FDS deployment [26, 28, 29, 60]. In the case of CompSE, EL, like SP, showed little change before and after stenting, with CR remaining within ±6% in all cases. This indicates that the “ease of flow” of blood was similar before and after FDS deployment, suggesting that blood continued to flow smoothly even after FDS deployment. In contrast, in the IncompSE cases, as with SP, EL increased after FDS deployment in all cases, with an average CR of 86.6%. These changes are thought to be caused by the impingement of blood flow on the FDS wires or by blood inflow vortices in the aneurysm resulting from IncompSE. In other words, the significant increase in EL compared to the CompSE cases suggests that the FDS, primarily designed to perform flow diversion, may essentially compromise blood flow due to IncompSE.

## 5 Limitations

Although our study revealed possible hemodynamic effects due to IncompSE, some potential limitations should be considered regarding the artery and aneurysm models, the geometry of IncompSE, and computational conditions. First, this study was conducted based on the basic geometry of artery and aneurysm. The simple models represent a large aneurysm located on the siphon part of the ICA, as the FDS is often used to treat large aneurysms in this region, such as ICA‐cavernous aneurysms. Although we modeled three types of “artery and aneurysm” to reflect the spatial relations between the location of IncompSE and aneurysm neck orifice, we have not explored the broader variety of artery and aneurysm types and their relationships observed in clinical practice. We acknowledge that factors such as the morphology and geometry of the parent artery, the aneurysm neck width, and the aspect ratio significantly influence aneurysmal hemodynamics. A more comprehensive investigation incorporating these factors would provide a deeper understanding of the hemodynamic effects of IncompSE. Future studies should address these anatomical variations to enhance the applicability of the findings. In addition, while various IncompSE patterns exist, including differences in the degree of expansion or its location, a single IncompSE model geometry was applied to reflect situations occurring on the proximal and distal sides of the aneurysm. Therefore, our present results cannot be directly generalized to all types of aneurysms or IncompSE situations. However, we believe that our findings provide an important perspective, suggesting that IncompSE might be a contributing factor to hemodynamic changes involved in incomplete occlusion or postoperative complications after FDS deployment. To address this limitation, future studies should incorporate patient‐specific geometries obtained from medical imaging to allow for a more accurate representation of individual clinical cases. The CFD analysis includes conventional limitations, such as the assumption of a rigid arterial wall, non‐patient‐specific boundary conditions, and the use of a Newtonian model for blood flow. Additionally, the steady flow simulation did not account for pulsatile blood flow, a simplification made to reduce computational costs and constituting a limitation. However, these methodologies have been reported to be reasonable simplifications for characterizing the basic trends of blood flow in aneurysms or cerebral arteries [61, 62, 63, 64, 65]. As suggested by previous studies, both steady and pulsatile flow simulations provide valuable insights into the fundamental characteristics of blood flow, and important dynamics can be understood with either approach [62]. The primary aim of this study is to reveal the potential hemodynamic changes caused by the occurrence of IncompSE. Therefore, these simplifications are reasonable for understanding the structure of the flow field and its changes. Future studies could address these limitations by incorporating a non‐Newtonian fluid model and an elastic arterial wall model to more accurately represent physiological conditions and further improve the accuracy of hemodynamic simulations. While steady flow simulations provide valuable insights, they do not fully capture the dynamic nature of blood flow. Therefore, future studies should incorporate pulsatile flow simulations to more accurately reflect time‐varying physiological conditions and to investigate the effects of IncompSE under more realistic scenarios. Another limitation is the lack of clear relationships between changes in the investigated parameters and treatment outcomes, including occurrence of complications (i.e., because the basic model was applied, we lack clear evaluation criteria to determine whether the present hemodynamic change will influence the occurrence of complications after the FDS deployment). Further detailed investigations in patient‐specific cases are needed to reveal the direct causal relationship between hemodynamic changes triggered by IncompSE and poor posttreatment prognosis. However, as demonstrated in this study, it is important to understand that, depending on the situation, the occurrence of the IncompSE might introduce potentially unfavorable hemodynamic changes, such as increased blood flow into the aneurysm or increased pressure on aneurysmal walls, when surgeons apply braided stents, including FDS.

## 6 Conclusions

In this study, we investigated the hemodynamic effects of IncompSE in FDS using multiple basic models that assumed different aneurysms located in the ICA siphon region. For each aneurysm model, CFD analysis was conducted for four patterns: before FDS deployment, CompSE, IncompSE on the distal side, and IncompSE on the proximal side. By comparing the hemodynamics across a total of 12 patterns, the following potential effects of IncompSE were identified.

- Insufficient flow diversion effect increases blood inflow into the aneurysm, preventing complete occlusion.
- WSS is elevated at the aneurysm neck and on the parent artery.
- IncompSE increases the pressure on the aneurysm wall, which may contribute to delayed rupture.
- IncompSE obstructs blood flow in the parent artery due to impingement on the FDS wires, resulting in increased EL.

## Ethics Statement

The authors have nothing to report.

## Conflicts of Interest

The authors declare no conflicts of interest.

## Data Availability

The data that support the findings of this study are available from the corresponding author upon reasonable request.
